# Methods for Evaluating Tibial Accelerations and Spatiotemporal Gait Parameters during Unsupervised Outdoor Movement

**DOI:** 10.3390/s24206667

**Published:** 2024-10-16

**Authors:** Amy Silder, Ethan J. Wong, Brian Green, Nicole H. McCloughan, Matthew C. Hoch

**Affiliations:** 1 Naval Health Research Center, San Diego, CA 92106-3521, USA; 2 Leidos, Inc., La Jolla, CA 92152, USA; 3 College of Health Sciences, University of Kentucky, Lexington, KY 40506, USA

**Keywords:** inertial measurement unit, military, ruck march, hiking, wearable sensor, GPS, GIS, data fusion

## Abstract

The purpose of this paper is to introduce a method of measuring spatiotemporal gait patterns, tibial accelerations, and heart rate that are matched with high resolution geographical terrain features using publicly available data. These methods were demonstrated using data from 218 Marines, who completed loaded outdoor ruck hikes between 5–20 km over varying terrain. Each participant was instrumented with two inertial measurement units (IMUs) and a GPS watch. Custom code synchronized accelerometer and positional data without a priori sensor synchronization, calibrated orientation of the IMUs in the tibial reference frame, detected and separated only periods of walking or running, and computed acceleration and spatiotemporal outcomes. GPS positional data were georeferenced with geographic information system (GIS) maps to extract terrain features such as slope, altitude, and surface conditions. This paper reveals the ease at which similar data can be gathered among relatively large groups of people with minimal setup and automated data processing. The methods described here can be adapted to other populations and similar ground-based activities such as skiing or trail running.

## 1. Introduction

The field of biomechanics is witnessing a surge in the utilization of wearable sensors, partly attributable to enhancements in battery life and internal memory capacity. While these advancements help facilitate the collection of large data sets in real-world conditions, using wearable sensors for research requires either reliance on commercial software or proficiency in mathematical and computational skills (e.g., [1,2]). Moreover, the sheer size of these datasets that are collected over long periods of time mandate the use of fully automated programs for analysis, thereby adding an additional constraint that accurate results can only be obtained through reliable quality checks and data processing, preferably at a reasonable computational cost. These requirements are not trivial. A review conducted by Edwards et al. (2023) [3] revealed that out of 78 manuscripts that used wearable sensors to investigate tactical athletes in the real world, none analyzed their data at a granular enough level to report how variables like physiological fatigue, terrain variations, or distance travelled may have affected movement and performance over time.

Accurately measuring body segment kinematics and kinetics using inertial measurement units (IMUs) requires the sensor to align with the segment’s anatomical axis(es) of interest. This is not always done. For example, when estimating tibial accelerations, many researchers falsely assume the IMU coordinate system is aligned with the tibia [4,5]. Others mitigate orientation sensitivity by only reporting the norm, or magnitude, of the tri-axial acceleration [6,7]. While useful in many ways, reporting the magnitude of acceleration precludes assessment of accelerations contributing to compressive and bending forces. IMUs can also be purchased with associated software that includes automated calibration and processing algorithms to facilitate the estimation of joint angles or segment orientation (e.g., [8,9]). These products often come at a premium price and necessitate specific sensor placement and calibration protocols in accordance with the software. This is both time-consuming and reduces accuracy when done improperly. Further, in outdoor environments, often characterized by low visibility and varying weather conditions, attempting consistent sensor placement and alignment becomes impractical. It is not, however, necessary to make any assumptions with regards to IMU placement or orientation. Various studies have successfully employed simple, dedicated calibration routines that determine sensor orientation with respect to a segment’s anatomical axis [10,11,12,13,14,15,16]. For applications that involve able-body walking, this can be done through a simple calibration method taken from periods of standing and walking. This method has been successfully implemented in laboratory setting using a dedicated bout of standing and walking [9], but the validity of this concept has not been tested during unsupervised outdoor walking.

During most indoor and outdoor data collections, the researcher is typically present to observe the participant and record relevant notes. This process is gradually becoming less necessary, particularly in team sports where athletes’ movements are tracked using markerless motion capture, radio frequency identification (RFID) tags, or a combination thereof. Data scientists can use this information to estimate total distance travelled, exertion levels, and, through advanced modeling, musculoskeletal loading, and team dynamics. For applications such as military training, long distance running, or community activity tracking, it is not practical to monitor and follow participants the entire time. In these situations, an individual is either outfitted with IMUs or accelerometers to measure musculoskeletal loading [17], or more commonly, they are outfitted with one of the numerous commercially available wrist worn devices that can measure heart rate, distance travelled, and a summary of total activity. While IMUs and wrist worn devices are individually beneficial, data from IMUs and wrist worn sensors can be combined to provide valuable synchronous information on tibial accelerations and spatiotemporal gait characteristics. This provides exclusive information to help researchers evaluate how changing external factors (e.g., terrain, ground-slope) affect fatigue, musculoskeletal injury risk, and performance in remote environments without the presence of research staff.

Numerous well-conducted studies on military load carriage have shown that movement patterns are affected by incline, speed, as well as the magnitude and distribution of load (e.g., [18,19,20,21,22,23,24]). Yet, all of these studies were conducted for relatively short periods of time in a laboratory. This limitation is important because prolonged load carriage [25] and fatigue inducing exercises [26] have both been shown to induce changes in gait kinematics when walking with 15 kg [26] or 30 kg [25] backpacks. Advancing on the work of these two studies, Bloch et al. (2024) used IMUs to show that spatiotemporal gait patterns and trunk lean change over the course of an outdoor 12–15 km loaded hike with ~40 kg load [27]. The work by Bloch and colleagues is important for many reasons, in part because laboratory studies cannot replicate operational training hikes that include factors such as changing environmental conditions, terrain features, and interaction with other unit members.

There is a pressing need to develop universal methods for accurately measuring and analyzing movement across diverse remote and unsupervised outdoor settings, devoid of dependency on commercial software or the need to have advanced mathematical and computational expertise. The purpose of this paper is to describe methods to synchronously collect IMU and GPS watch data with minimal participant interaction. We describe the sensor settings and data collection procedures used to minimize participant interaction while ensuring adequate data quality. The automatic processing methods produce results with high temporal and spatial resolution for tibial accelerations, spatiotemporal gait metrics, heart rate, gait speed, and terrain features during military ruck hikes. We show examples of how the utilization of these methods will ultimately improve our understanding of the factors that affect performance or increase injury risk, and how changes in performance over long distance training hikes can be used to guide the development and understanding of training programs.

## 2. Materials and Methods

**Figure 1:**
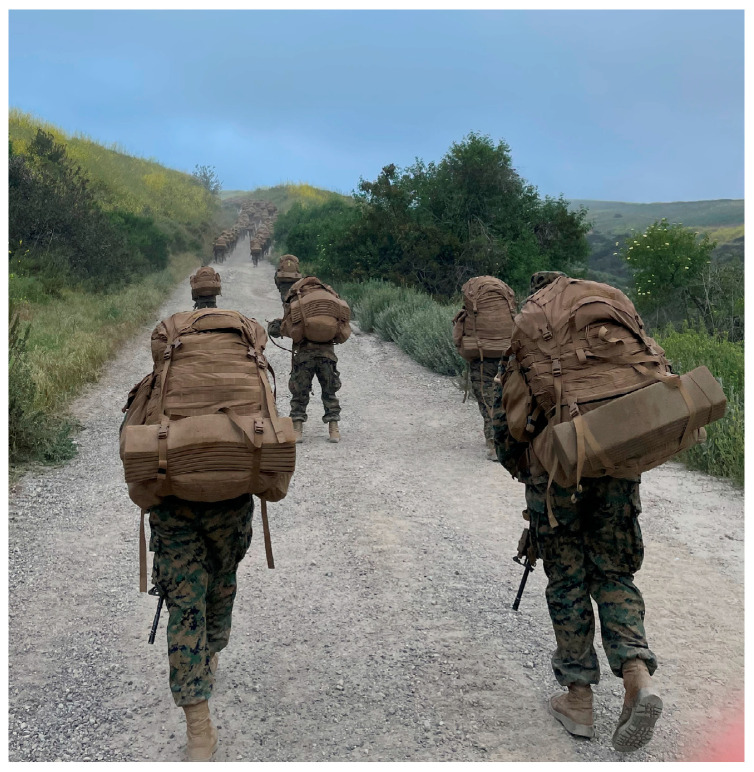
Participants hiked on primarily fanglomerate surface (hardtop shown above) while carrying a loaded ruck and weapon. (1) Automated calibration of the IMU relative to the tibia assumed primary motion of the leg was flexion-extension. The extent to which this is true varied among participants, as is demonstrated by Marines pictured on the left and right of the picture. (2) Participants often stopped to rest during their hike, as is demonstrated by the Marine pictured center. Periods of rest were excluded from analysis, by removing movements less than 0.45 m/s. Picture courtesy of Amy Silder.

A total of 218 Marines (157 M/61 F, mean ± standard deviation, 20 ± 2.4 y, 1.73 ± 0.10 m) provided written informed consent to participate in this study, in accordance with a protocol approved by the University of Kentucky Institutional Review Board (Protocol number UK 62029). Data were collected during a series of outdoor training hikes that were 5–20 km in length over a predominantly fanglomerate surface. Participants completed the hikes in full combat uniform (participants’ mass in uniform, 76.3 ± 10.7 kg) while carrying a loaded ruck (29.1 ± 6.1 kg) and weapon (3.3 kg) (Figure 1).

### 2.1. Data Collection

#### Sensor Settings and Data Collection Procedures

Prior to hiking, each participant was equipped with a Garmin^TM^ Solar Instinct 2 GPS watch (Garmin, Schaffhausen, Switzerland) secured tightly on the posterior wrist, along with one XSens DOT IMUs (XSens, Enschede, The Netherlands) strapped securely to each anterior-medial tibia, directly above the boot. Tri-axial accelerometer and angular velocity data were recorded in the local sensor coordinate system. Onboard analog data were sampled at 800 Hz for all data collected as part of this study, and onboard analog-to-digital processing downsampled the data to 30 Hz for the first 72 participants and 60 Hz for the final 146 participants. The increase in reported frequency was enabled after a hardware and firmware update. We used Xsens’ dynamic setting for all data collections (i.e., before and after the firmware update), which enabled these relatively low collection frequencies without causing clipping in peak accelerations [28].

To minimize time spent interacting with the participants, recording was started on the IMUs prior to meeting with the participants. In-person setup time involved securing the IMUs, ensuring proper fit of the GPS watch, and ensuring each participant started recording on their GPS watch. This took approximately 1–2 min per participant. After setup, participants returned to their individual unit to prepare for the hike, completed the training hike under the supervision of their unit commander, and met with researchers after the hike to remove the sensors.

### 2.2. Data Processing

#### 2.2.1. Data Extraction and Synchronization

Data from the GPS watch and IMUs were transferred to a computer via a direct cable. Data from the GPS watches were saved as training center XML files, and data from the IMUs were saved as comma-separated value (CSV) files. XML files were imported into MATLAB using the function, *xmlread* (Version R2023b, The MathWorks, Natick, MA, USA). Custom code was thereafter used to extract positional and heart rate data at each time point. CSV files were imported into MATLAB using the MATLAB function, *readmatrix*. IMU data included time, three-dimensional acceleration and angular velocity.

As described in Section Sensor Settings and Data Collection Procedures, the GPS watch and IMUs were not started at the same time. Global time for the GPS watches were obtained from atomic clocks within the communicating satellites. Global times for the IMUs were established using the Network Time Protocol, obtained directly from the phone or device that initiated the start of recording. We synchronized data from all three sensors, to the nearest second, using these global time stamps. All data prior to the start of the device that started last (i.e., GPS watch) was eliminated. Although data prior to the start of the hike was saved, it was classified as non-movement, as described in Section 2.2.4 below.

#### 2.2.2. Orientation of IMU in Tibial Reference Frame

IMU orientation relative to the tibia anatomical reference frame was determined using gravitational acceleration during quiet standing and the first principal component of angular velocity during walking. Our methods employ the same mathematical principals and calibration procedures recommended by the XSens MTw Awinda system [9]. These calibration routines involve standard protocols such as static standing, stationary periods, and joint flexion-extension movements [10,11,12,13,14,15,16].

The long axis of the tibia was defined using the gravity vector during quiet standing. We used the MATLAB function, *ischange*, to automatically identify the first stint of data during which the x, y, and z acceleration variances were less than 0.005 m/s^2^ between change points, with a threshold equal to 1. The first stint of data was used to ensure the participant was not lying or sitting, which often occurred during rest periods during the hike. Note, although we used a changepoint threshold value of 1, values between 1 and 10 were also suitable and did not affect the algorithm’s ability to detect periods of non-movement. A variance of 0.005 m/s^2^ was iteratively chosen with the goals of minimizing participant movement while also ensuring at least 1.00 s of standing was identified for all 218 participants. Acceleration data from this stint of quiet standing was averaged and the vector norm was used to define z+ in the distal-to-proximal direction of the tibia.

The medial-to-lateral axis of the tibia was estimated using the first principal component of angular velocity during the longest continuous bout of walking, taken during the first 60 min of the hike. Note that walking was defined as any movement between 0.45–1.79 m/s [29]. Assuming primary motion of the leg during walking is flexion-extension, the first principal component of angular velocity is the flexion-extension axis. As such, the vector norm of the first principal component was used to define y+ in the right lateral direction. We used walking data from only the first 60 min of the hike to minimize the chance that changes in gait due to fatigue would influence the accuracy of our calibration.

The cross product of y and z were used to define x+ in the anterior direction, and the cross product of x and z was subsequently computed to redefine y+ and ensure mutual orthogonality. These vectors determined the fixed rotation matrix between the local sensor and tibial coordinate systems [11].

#### 2.2.3. Validation of IMU Orientation

The method to calibrate sensor orientation described above automatically detected standing and walking phases and required no interaction with the research participants. This method assumes that these phases were sufficiently similar to a dedicated calibration routine consisting of standing and knee joint flexion-extension. The automated calibration method was therefore evaluated using 50 randomly chosen participants who completed a dedicated calibration routine. We compared the absolute angle between the x, y, and z rotation vectors from the automated method with the rotation vectors determined from a separate dedicated calibration routine.

The dedicated calibration method was conducted similar to previous studies [12,14,15,16]. It involved approximately 5 s of quiet standing followed by 5 knee flexion extension tasks on each leg. Data from these periods were manually chosen using the MATLAB function, *ginput*, and the same mathematical methods as described in Section 2.2.2 above were used to estimate the rotation matrix between the local sensor and tibial coordinate systems. The error in the automated method was defined as the angle between the x, y, and z vectors between the automated and dedicated calibration methods, computed as the arccosine of the dot product between each of the two vectors.

#### 2.2.4. Tibial Accelerations and Spatiotemporal Gait Characteristics during Movement

Activity (e.g., non-movement, walking, running) throughout the hike was classified using speed, which was estimated as the distance travelled divided (described below in Section 2.2.5) by the time between GPS time-points. Walking was defined as movement 0.45–1.79 m/s [29] and running as movement 1.80–5.36 m/s. Although the average walking speed among healthy adults is approximately 0.82 m/s [29], we chose to include speeds as low as 0.45 m/s to account for participants who walked slower than normal, particularly on steep inclines. Conversely, while many healthy young adults can run at speeds exceeding 5.36 m/s, we established this as our upper cutoff for running to exclude instances where some participants ran faster than 5.36 m/s down very steep sections of the hike. It is important to note that these ranges only act to categorize the data for visualization and post-hoc analyses and can be changed in response to a different research or operational question.

The approximate time of each foot contact was estimated by finding the minimum jerk of the resultant acceleration using the MATLAB function, *localmin,* assuming maximum cadence of 180 steps/min [30]. These timepoints were used to calculate cadence and extract corresponding peak vertical and posteriorly directed accelerations. Step length was subsequently estimated as the product of speed and step frequency.

#### 2.2.5. Terrain Features

Ground aspect and slope were estimated using a publicly available high resolution LIght Detection and Ranging (LIDaR) scan of the research area with nominal pulse spacing of 0.7 m [31]. This map was imported into ArcGIS Pro software (Version 3.2, Esri, Redlands, CA, USA). We then used the ArcGIS Raster Functions of *Aspect* and *Slope* (located under the Surface tab) to generate slope and aspect rasters of the entire region within the LIDaR scan. These rasters were then exported using the ArcGIS function, *Export Map*, to create Tiff images of the research area. The images were exported at 8-bit, with a latitude-longitude resolution of approximately 1.2 × 10^−3^ m.

**Figure 2:**
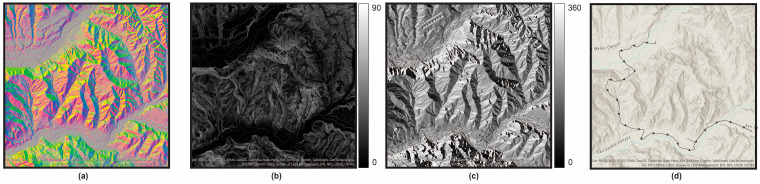
(**a**) A high resolution publicly available Light Detection and Ranging (LIDaR) scan [31] of the research area was used to calculate (**b**) slope in degrees and (**c**) aspect in positive degrees measured from clockwise North. (**d**) This was combined with the azimuth between successive time points to estimate gradient. The curved line in the figure represents 2453 azimuths for one participant. Arrows indicate the direction of travel and were subsampled for visualization purposes.

The slope and aspect files were then used in conjunction with the participant’s location data throughout the hike to estimate their distance travelled as well as the inclination angle, or gradient, on which the participant was walking or running. To do this, we computed the distance travelled between each time point for each participant using the MATLAB function, *azimuth*, meaning the great circle bearing, expressed in degrees clockwise from North, ranging from 0 to 360 degrees (Figure 2d). For extremely short distances, such as those considered here, the azimuth can be visualized as a vector whose initial point is the latitude-longitude location of the participant at any given time point and whose terminal point is the next latitude-longitude location of that same participant. The azimuth was therefore also used to determine each participant’s direction of travel between successive time points. The participants’ location at each time point in the GPS file along with the associated azimuth were then mathematically “overlaid” onto the slope and aspect raster data to geometrically estimate the gradient and side-slope between each successive time point. Figure 2d is a representation of this process, overlaid onto a surface map for visualization purposes.

## 3. Results

### 3.1. Validation of IMU Orientation Relative to Tibia Reference Frame

IMU orientations obtained from the automated calibration routine were compared to IMU orientations obtained from the dedicated calibration routine by computing the absolute angle between the vectors representing the x, y, and z elements of each rotation matrix, as described above in Section 2.2.3. The average difference between vectors among all 50 randomly chosen participants was 0.107 ± 0.093 rad, 0.087 ± 0.055 rad, and 0.093 ± 0.107 rad for the x, y, and z directions, respectively. This would result in average errors of 1–2%, compared to the dedicated calibration routine.

Importantly, the error for 6 of the participants was substantially higher than for the remaining 44 participants, particularly in the flexion-extension axis (y-direction). The average error among these 6 participants was 0.129 rad, 0.274 rad, and 0.078 rad for the x, y, and z directions, respectively. These errors would result in a maximum 4% error (y-direction) compared to the dedicated calibration routine. During the automated calibration process, the *y*-axis is defined using the first principal component of angular velocity during walking, thereby assuming that walking occurs exclusively in the sagittal plane. We hypothesize these errors were caused by greater than normal medial rotation of the tibial of certain individuals during swing phase (Figure 1). While this error is substantially higher than for the remaining participants, it is important to consider the tradeoff between the additional time and effort required to perform a dedicated calibration routine and the increased error for a small number of participants.

### 3.2. Speed

**Figure 3:**
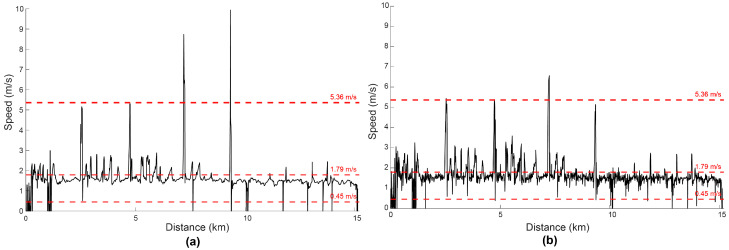
Speed of one representative participant as a function of distance travelled. Walking was defined as movement 0.45–1.79 m/s [29] and running as movement 1.80–5.36 m/s: (**a**) Taken directly from the GPS watch data; (**b**) Estimated as the distance travelled between two successive time points, divided by the time elapsed between these two successive time points.

The GPS watch used in this study exports speed at every time point during recording. We chose to estimate speed separately because the speed values reported by Garmin^TM^ were not realistic, as shown from a representative participant in Figure 3a, who did not travel at speeds of ~9–10 m/s. The details of how speed was estimated from the watch are proprietary and, therefore, not disclosed. As a result, we estimated speed between any two successive time points as the distance travelled (described in Section 2.2.5), divided by time elapsed between the same two successive time points (Figure 3b).

### 3.3. Other Outcomes

In addition to positional data, the Garmin^TM^ Instinct 2 watch measured heart rate and weather data.

Watches were worn snug and on the top of the wrist to help ensure accurate heart rate data. The average heart rate during hiking was 151 ± 14 bpm, with a maximum of 187 ± 11 bpm.

Weather data was obtained from the nearest weather station and taken the start of the recording. Data include temperature, sky conditions, and precipitation (temperature, sky condition, precipitation).

### 3.4. Data Summary

**Figure 4:**
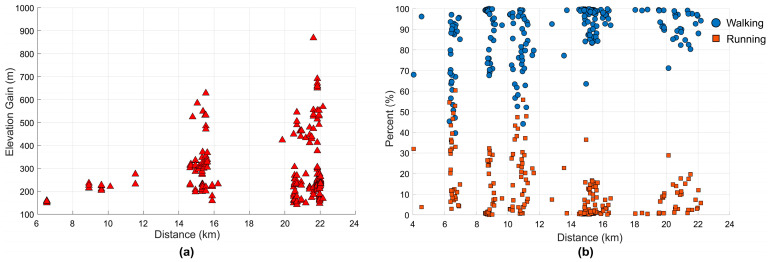
(**a**) Elevation gain and (**b**) the percent of the distance travelled that was spent walking (0.45–1.79 m/s [29]) and running (1.80–5.36 m/s) for the 218 participants in this study.

The 218 participants enrolled in this study walked a total of 2858 km and took an es-timated 10 million steps. The hikes tended to be 5 km, 10 km, 15 km, or 20 km in length with an average elevation change, respectively, of 180, 240, 374, 558 m (Figure 4a). Of the total distance travelled, 72.5% was classified as walking (1.33 ± 0.07 m/s), and 9.3% was classified as running (2.26 ± 0.23 m/s). The shorter hikes tended to be completed faster and with a greater percentage of time running compared to walking (Figure 4b). Although not directly reported, these data and results directly enable investigation into numerous inter-related other factors related to long distance military load carriage, including changes in gait speed, total weight carried, weight carried as a percentage of body weight (39 ± 9%), sex, soil and surface conditions, angle of attack, heart rate (i.e., physiological effort), and/or temperature.

## 4. Discussion

This manuscript introduces methods for measuring gait patterns with high spatial and temporal resolution over extended periods in unsupervised outdoor conditions. Dismounted warfighters face demanding physical workloads, often operating in adverse conditions such as darkness and inclement weather, while carrying heavy equipment. Historically, military load carriage policy decisions have primarily referenced the US Army Load Carriage Decision Aid (LCDA), a comprehensive tool encompassing metabolic equations accounting for various factors like terrain [32,33], walking speed [34], gradients [35], modern backpack design [36] and load distribution [37,38]. Monitoring warfighter movement solely through wrist-worn sensors overlooks crucial time-dependent factors including physiological loading information that may change throughout the course of a hike. Similarly, measuring warfighter movement in controlled laboratory conditions overlooks crucial factors that exist during real-world training, such as weather, terrain, and variability in load configuration. The data collection and processing methods presented in this manuscript not only address these limitations but also significantly reduce the time required to gather essential information, enabling more effective decision-making for military operations and injury prevention strategies.

Research on the biomechanics of long-duration outdoor movements has primarily focused on running athletes (e.g., [39,40]). It wasn’t until recently that a study by Bloch et al., (2024) measured changes to gait kinematics during an extended ruck hike. Alternative methods for recording and analyzing military ruck hikes have predominantly relied on either IMUs [27] or a GPS watch [41]. Our study builds upon this previous work by integrating the use of both an IMU and GPS watch. We placed the IMU along the shank to collect and analyze tibial accelerations and combined these data with synchronized location information. Doing this allowed us to measure spatiotemporal gait patterns, tibial accelerations, and heart rate throughout the duration of the hike. We paired these biomechanical gait outcomes with high resolution environmental features to enable a deeper understanding of the effects of hike duration, terrain, ground cover, and other geospatial features on gait biomechanics. In this manuscript, we described and demonstrated the feasibility of our methods to collect large volumes of data with minimal participant interaction. Further analysis of this dataset promises significant advances in understanding injury risk and performance during military training hikes. Additionally, our methods can be readily implemented or easily adapted for other populations and environments.

Musculoskeletal injuries are ubiquitous in the military [42]. Unlike long distance running, the diverse nature of military activities makes predicting and monitoring overuse injuries even more challenging. Factors such as gait speed, total weight carried, weight carried as a percentage of body weight, heart rate, and sex have all been investigated to various extents in laboratory conditions over relatively short periods of time (e.g., [19,20,21,22,23,24,43,44,45]). Other studies have found changes in gait kinematics during load carriage after walking for relatively long distances on a treadmill [25], or following strenuous exercises [26]. Although tibial accelerations are generally not well correlated with ground reaction forces [21,46] and are not strongly linked to increased risk of fatigue fractures in runners [47], increased tibial accelerations have been found after a fatigue-inducing treadmill run [48]. In this context, measuring tibial accelerations during long-distance training hikes can provide valuable insights into their potential impact on injury risk in military populations.

The impact of long-distance ruck hikes on overall gait patterns remains largely unexplored [27]. This is not trivial, as collecting data without interfering with training requires minimal setup time and minimal interaction with participants once training has begun. The methods used in our study required only a few minutes of setup and were paired with automatic processing that accounts for sensor synchronization, sensor alignment, and periods of non-movement. The methods can be adapted for use with other IMUs, accelerometers, and Global Navigation Satellite System (GNSS) receivers. For example, we chose to use XSens DOT IMUs due to their small size, cost-effectiveness, and accuracy over long durations. However, since the start of our research study, wearable sensor technology has rapidly advanced, and many other commercial and custom-built options have emerged that now meet or exceed the capabilities of those available at the study’s inception. Further, the integration of high-resolution topographical data with gait and movement information opens the door to explore the effects of vegetation, ground surface, sun exposure, and other environmental factors that may provide invaluable insights into musculoskeletal loading, fatigue, and overuse injury risk.

There are certain limitations and improvements to this study that should be considered. For example, one motivating factor in using the methods presented in this paper was to diverge from commercial black-box algorithms. While this was generally achieved, we were not able to directly use raw speed and altitude data from the GPS watch. It is unknown how these outcomes were estimated, but they differed from our own estimations using GPS positional data combined with high resolution, publicly available topography (Figure 2). Consumer-grade GPS watches are generally considered to be accurate to approximately 3 m [49] in open environments such as where these data were collected, but future efforts should nevertheless consider the tradeoff between ease of use for wearing and recording on a GPS watch versus an alternative sensor capable of measuring more accurate latitude-longitude. Second, while we synchronized the start time of all devices to the nearest second, a non-precise synchronization of sensors precludes any assessments into phases of the gait cycle (e.g., double support), where precise relative timing between limbs is required. Finally, we used the peak jerk of the magnitude of acceleration to approximate foot contact events and constrained foot contacts to occur at a maximum of 180 steps/min [30].This is a reasonable assumption for our application or any application looking to assess typical walking and running patterns, but it also removes fast gait which may be desired or necessary in certain environments.

In conclusion, this manuscript presents a significant advancement in the field of monitoring spatiotemporal gait patterns and tibial accelerations during extended ruck hikes over varied remote terrain. By using methods that consider continuous monitoring of tibial accelerations along with heart rate and environmental terrain, our study addresses a critical gap in understanding the complexities of musculoskeletal loading, fatigue, and injury risk during prolonged hiking events. Moreover, our approach enables data collection without disrupting training routines, thus offering a practical solution for long-term monitoring. Overall, this study lays a foundation for future research aimed at better understanding and mitigating musculoskeletal injuries among military personnel engaged in extended outdoor activities.

## Data Availability

The dataset and code presented in this article are not readily available because the data are part of an ongoing study. Requests to access the dataset and code should be directed to the corresponding author.

## Disclaimer

AS, EW, BG and NH are employees of the U.S. Government. This work was prepared as part of their official duties. Title 17, U.S.C. §105 provides that copyright protection under this title is not available for any work of the U.S. Government. Title 17, U.S.C. §101 defines a U.S. Government work as work prepared by a military service member or employee of the U.S. Government as part of that person’s official duties. This work was supported by the Office of Naval Research under work unit no. 62333. The views expressed in this research are those of the authors and do not necessarily reflect the official policy or position of the Department of the Navy, Department of Defense, nor the U.S. Government. The study protocol was approved by the Naval Health Research Center Institutional Review Board in compliance with all applicable Federal regulations governing the protection of human subjects. Research data were derived from an approved Naval Health Research Center and University of Kentucky Institutional Review Board protocol, number UK 62029.
